# Characterization of a typical urban soil in terms of natural radionuclide content. The case study of a university campus

**DOI:** 10.1016/j.heliyon.2024.e37145

**Published:** 2024-08-30

**Authors:** Serpil Aközcan Pehlivanoğlu, Simona Mancini, Selin Özden, Michele Guida, Mariarosaria Falanga

**Affiliations:** a Kirklareli University, Faculty of Science and Literature, Department of Physics, Campus of Kayali, 39100, Kirklareli, Turkey; b Laboratory Ambient and Radiations (AmbRa), Department of Information and Electric Engineering and Applied Mathematics (DIEM), University of Salerno, via Giovanni Paolo II, 134 84084, Fisciano, Italy; c Department of Information and Electric Engineering and Applied Mathematics, University of Salerno, via Giovanni Paolo II, 134 84084, Fisciano, Italy; d Istituto Nazionale di Geofisica e Vulcanologia, Sezione di Napoli–Osservatorio Vesuviano, via Diocleziano, 328 - 80124, Naples, Italy

**Keywords:** Urban soil, HPGe, γ-ray spectrometry, Natural radionuclides, Gamma dose

## Abstract

A first comprehensive survey was carried out in a university campus in Italy in order to investigate in terms of natural elements an area where medium-high values of natural radiation are expected because of its peculiar geological features. The content of terrestrial radionuclides in 20 topsoil samples from the campus was determined with the aim to provide an important database of the soil characteristics.

^226^Ra, ^232^Th, and ^40^K concentrations were analysed by High Purity Germanium (HPGe) gamma-ray spectrometer in order to determine the background levels of natural radionuclides characteristics of the original area. The mean concentrations of radionuclides in the investigated soil samples ranged from 58.95 ± 4.20 to 158.05 ± 19.95 Bq kg^−1^ for ^226^Ra, from 72.28 ± 7.61 to 146.00 ± 22.27 Bq kg^−1^ for ^232^Th and, for ^40^K, from 550.76 ± 33.24 to 1367.50 ± 18.73 Bq kg^−1^. The radiological hazard indices, including radium equivalent activity, external hazard index, annual effective dose, absorbed dose rate, lifetime excess cancer risk, were also evaluated and compared with global averages, revealing values above the worldwide ones. Finally, a spatial modelling methodology of the site-specific radionuclides levels as graphical tool for the monitoring of the potential land redevelopment of urban soils was proposed.

## Highlights

• ^226^Ra, ^232^Th, and ^40^K gamma spectrometry measurements.
• Assessment of the impact on human health and statistical analysis of the estimated parameters.
• Spatial distribution of the background levels of ^226^Ra, ^232^Th, and ^40^K in an urban soil.

## 1 Introduction

There are natural and man-made sources of radiation in the environment to which there is always exposure. Natural radiation sources represent the most important part of the total radiation exposure in the environment. The primary source of external radiation for humans is naturally occurring radioactive materials (NORMs), which are typically present at various levels in all environments [1]. NORM includes long-lived radioactive elements such as uranium, thorium and potassium and any of their decay products such as radium and radon [2]. Soil is the main source of all life-supporting substances, directly or indirectly, and is also the main distributor of the natural background radiation dose to which humans are exposed. Soil, which is the upper part of the earth's crust formed by the deformation of the rock as a result of physical and chemical processes, contains not only organic and inorganic matter, but also naturally occurring radionuclides such as Uranium (^238^U), Thorium (^232^Th), Potassium (^40^K) and their decay products. These isotopes are influenced by different geological and geographical factors since their distribution in soil generally depends on the characteristics of the parent rock and soil, so varying from one site to another. In general, relatively increased radioactivity is associated with igneous rocks and decreased with sedimentary rocks. Igneous rocks, that is, sialic rocks (especially granitoids), contain significantly higher concentrations of natural radionuclides than ultramafic and mafic rocks. However, there are some exceptions, such as some shales and phosphates that show relatively high radionuclide content [3,4].

The Italian peninsula has a unique intricate geological framework as result of several geodynamic events associated with the opening and closure of the Tethyan Ocean [5]. The geological structure of the Italian territory comprises recent volcanic rocks, granitic areas, and high permeability soils located in the vicinity of seismic faults. Among the Italian regions, Campania, a region located on the south-western part of the Italian peninsula, exhibits all those features in its territory characterized by the presence of volcanic lithotypes and sediments [6,7]. In this kind of areas, where medium-high levels of radon potential from soil are expected because of the geological features deeper investigation could be required not only for radiation protection purposes but also in the contexts of the characterization of the urban soil. The determination of the contents of natural radionuclides in soil could be intended as a fingerprint of the characteristics of the original soil important for the monitoring of topsoil redevelopment as consequence of the human and construction activities in urban environment. The characterization of such soils is not only relevant for a direct consequence on human health but also, in an indirect way, because they can play a role in climate change. Indeed, soils are complex systems made of plants, microorganisms, and rocks and they can be effective in the reduction of atmospheric CO_2_ and carbon sequestration. We remind that the atmospheric carbon dioxide is a major greenhouse gas resulting from global natural and human activities and has a significant impact on global warming. The presence of specific radionuclides in soil can affect the growth of plants and, thus, intervene in the radiative balance [8].

In light of the previous considerations, this research is devoted to the characterization of urban soils in a context where radiation protection is crucial. Therefore, this work presents the results of a first comprehensive study of the area of the campus of the University of Salerno, which can be considered as representative of urban soil frequented by staff, students and researchers. The present research is aimed to.

i) Determine the ^226^Ra, ^232^Th, and ^40^K activity concentrations in soils;
ii) Evaluate the radiological hazard indices and radiation doses received by people living in the area;
iii) Compare the results with international average values and threshold values defined by regulation;
iv) Propose a spatial distribution modelling methodology for the characterization of urban soils in terms of contents of natural radionuclides characteristics of the area.

In the ‘Materials and methods’ section all the phases and techniques used for the determination of the ^226^Ra, ^232^Th, and ^40^K activity concentrations in soils is described as well as the calculation of the international radiological hazard indicators for the assessment of the health risk, in terms of doses received by people living in the area. In section 3 results of the radiological hazard indices and radiation doses have been graphically represented and compared with international mean values and thresholds and a spatial distribution modelling methodology have been proposed as possible tool to implement by authorities for the visual characterization of the urban soils features in terms of contents of natural radionuclides. Future developments and improvements have been discussed in the conclusion section, then.

## 2 Materials and methods

### 2.1 Sample collection, preparation and analysis by gamma spectrometry

**Fig. 1:**
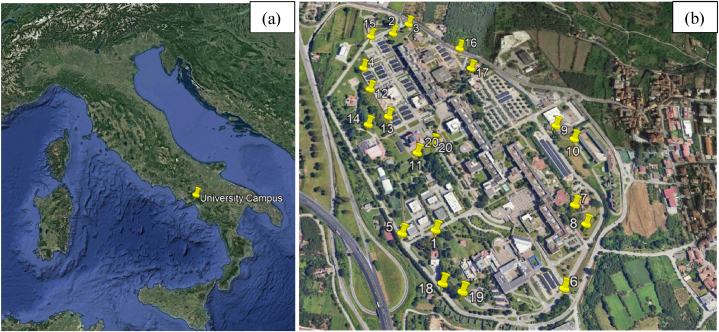
(a) Localization of the study area in the Italian Peninsula; (b) aerial view of the campus of Fisciano (Campania region, Italy) with evidence of the station measurements (@google earth, 2023).

The investigated area is the campus of the University of Salerno, located in the Campania region, south of Italy. The University of Salerno includes various campuses spread across the territory. The main one is located in the municipality of Fisciano, in the Irno valley. Settled on an area of 1,200,000 m^2^, it houses the majority of the facilities (canteen, theatre, police office, swimming pool, post office, students’ premises, etc) (Fig. 1). The area belongs to the Alto Irno-Solofrana territorial sector (Fig. 1(a)), homogeneous in terms of geological, morphological and anthropization conditions, and whose lithological formations, as can be observed from the map of the Radon Prone areas of the province of Salerno, has a medium-high level of radon potential from soil [7] which could represent a potential cause of the “Indoor Radon” problem and so interesting from a radioprotection point of view.

The territory is essentially covered by river sediments interspersed with pyroclastites deriving from the eruption of the Vesuvius volcano in the nearby Neapolitan area. The sedimentary succession, of which the valley is made up, is covered by a thin thickness of soil, which is found underneath by fluvial and pyroclastic deposits. This sequence extends for a thickness of 20–40 m and is supported by bases made up of volcanic tuffs. The area of our interest, corresponding to the alluvial fan on which the Campus extends, is characterized by an alternation of alluvial deposits with different depositional periods. At the ground level, there is the presence of vegetal soil which rests on a succession of horizons made up of alternations of gravel and silty sand, generally of a pyroclastic nature, which in turn overlies a layer of alluvial fan gravel (104m from the ground level) and one of ancient alluvial deposits (185m from the ground level) [6].

In order to model the spatial distribution of the content of ^226^Ra, ^232^Th, ^40^K, to characterize the area in terms of natural environmental pollution, 20 sampling points along the campus perimeter were selected (Fig. 1(b)).

Surface soil samples were collected from the 20 selected points. The soil samples were collected from a depth of about 0–10 cm. In the laboratory, soil samples were transferred to an oven at 105 °C until no detectable change in weight was noticed. Thereafter, they were grinded and sieved by using a 2 mm mesh screen to get fine homogenous powder samples [9]. The soil samples were sealed and stored 250 mL in cylindrical plastic containers for a minimum of 40 days to allow secular equilibrium between radium (^226^Ra) and its decay products before gamma spectrometric analysis.

The activity concentration of radionuclides in the samples was measured by using an HPGe gamma spectrometer (ORTEC, USA), with a relative efficiency of 70 % and a resolution of 2.0 keV for a gamma line of ^60^Co at 1332 keV. The calibrations were made using a standard mixed source with an energy range of 47–1836 keV (Eckert & Ziegler Isotope Products Laboratories) including known activity levels of ^210^Pb, ^139^Ce, ^109^Cd, ^241^Am ^57^Co, ^113^Sn, ^203^Hg, ^85^Sr, ^88^Y, ^137^Cs, and ^60^Co peaks.

All samples were counted for 86400s. Background intensities were also obtained under the same conditions before and after the measurement of the samples. GammaVision-32 software was used for the spectra collection and analysis of the spectra were carried out by Maestro (ORTEC GEM70P4-95) software. The activity concentration of ^226^Ra was determined from ɤ-ray lines at 351.9 keV for ^214^Pb and 609.3 keV for ^214^Bi, respectively. The content of ^232^Th was obtained from photo peaks of ^228^Ac at 911.2 keV and ^208^Tl at 583.1 keV. The activity concentration of ^40^K was determined using an ɤ-ray line at 1460.8 keV [10,11]. The following equation (Eq. (1)) was used to calculate the activity concentration of each radionuclide:

(1) $$A\left(Bq\;{kg}^{-1}\right)=\frac{N_{R}}{ɛ\;.\;m\;.{\;I}_{ɤ}}$$

where *A (Bq kg*^*−1*^*)* is the activity concentration of a radionuclide in the surface soil sample, N_R_ is the net ɤ counting rate, ɛ is the detection efficiency in the gamma energy of interest, *m* is the mass of the measured sample and I_ɤ_ is the ɤ-ray emission probability.

### 2.2 Radiological hazard parameter calculation

The outdoor and indoor absorbed dose rates (D_out_ and D_in_) were calculated by Eqs. (2), (3)). The outdoor absorbed gamma dose rate, *D*_*out*_ (nGy h^−1^) in air through terrestrial ɤ radiation at 1 m above the ground, has been calculated from the ^226^Ra, ^232^Th, ^40^K activity concentrations (*A*_*Ra*_*, A*_*Th*_*, A*_*K*_) [12]:

(2) $$D_{out}=0.462A_{Ra}+0.604A_{Th}+0.0417A_{K}$$

Indoor absorbed dose rate (*D*_*in*_) due to the presence of ^226^Ra, ^232^Th, ^40^K in indoor environments estimated using the following relation [12]:

(3) $$D_{in}=0.920A_{Ra}+1.10A_{Th}+0.0810A_{K}$$

Outdoor and indoor annual effective dose equivalent (*AEDE*_*out*_ and *AEDE*_*in*_) values can be identified by Eqs. (4), (5)). *AEDE*_*out*_ (μSv y^−1^) and *AEDE*_*in*_ (μSv y^−1^), caused by exposure to NORMs in surface soil samples, was calculated using the following equations [10,13,14]:

(4) $${AEDE}_{out}\;\left(\mu Sv\;y^{-1}\right)=D_{out}\;\left(nGy\;h^{-1}\right)\times 8760\;\left(hy^{-1}\right)\times 0.2\times 0.7\;\left(Sv{\;Gy}^{-1}\right)\times 10^{-3}$$

(5) $${AEDE}_{in}\;\left(\mu Sv\;y^{-1}\right)=D_{in}\;\left(nGy\;h^{-1}\right)\times 8760\;\left(hy^{-1}\right)\times 0.8\times 0.7\;\left(Sv{\;Gy}^{-1}\right)\times 10^{-3}$$

where Dout and Din are the absorbed gamma dose rate (nGy h-1); 8760 corresponds to the hours per year; 0.2 is the outdoor occupancy factor, 0.8 is the indoor occupancy factor and 0.7 is the dose convention factor (Sv Gy-1).

The Radium Equivalent Activity (Raeq) was calculated by using Eq. (5) [15]:

(5) $${Ra}_{eq}\left(Bq\;{kg}^{-1}\right)=A_{Ra}+1.43A_{Th}+0.077A_{K}$$

which assumes that 370 Bq kg^−1^ of ^226^Ra, 259 Bq kg^−1^ of ^232^Th or 4810 Bq kg^−1^ of ^40^K have the same ɤ dose rate ($A_{Ra},A_{Th},A_{K}$ are the activities of ^226^Ra, ^232^Th, ^40^K, respectively). Since NORMs are distributed nonuniformly in environmental media *Ra*_*eq*_ is a proper parameter to compare activity concentrations in samples and, thus, it has been determined to compare the activity concentrations of NORMs.

To estimate the level of radiological risk due to NORMs, external exposure to NORMs the radiation hazard index, *H*_*ex*_ was calculated for the surface soil samples by Eq. (6) [16]:

(6) $$H_{ex}=\frac{A_{Ra}}{370}+\frac{A_{Th}}{259}+\frac{A_{K}}{4810}$$

*H*_*ex*_ must be below unity to avoid radiation.

The Excess Lifetime Cancer Risk *(ELCR),* giving the lifetime cancer risk probability from exposure to ionizing radiation in any population, was estimated by using Eq. (7) [17]:

(7) $$ELCR=AEDE\;\left(\mu Sv\;y^{-1}\right)\times DL\;\left(y\right)\times RF\left({Sv}^{-1}\right)\text{,}$$

where *AEDE* is the annual effective dose equivalent; *DL* is the average duration (70 years), and *RF* is the fatal risk factor (0.057) [18].

### 2.3 Principal component analysis

The Principal Component Analysis (PCA) is an important statistical tool in various research fields from geochemical applications [19], radiological analysis [20], evaluation of radioactive pollution sources [21] to seismic [22], and acoustic environments [23]. It is a well-established method for performing a linear mapping of the data to a lower dimensional space in such a way that the variance of the data in the low-dimensional representation is maximized [24]. In practise, PCA gives information on the dimensionality of the measures and on the information, they carry on (for further details, please refer to Refs. [25,26].

The PCA was carried out using IBM SPSS 25. This method groups related dependent variables into a series of components by calculating an eigenvalue for each group of variables [27]. Varimax orthogonal rotation was applied to identify variables. Varimax rotation allowed us to obtain a clear system as a result of eliminating invalid components.

## 3 Results and discussion

### 3.1 Activity concentrations of ^226^Ra, ^232^Th and ^40^K in soil samples

**Fig. 2:**
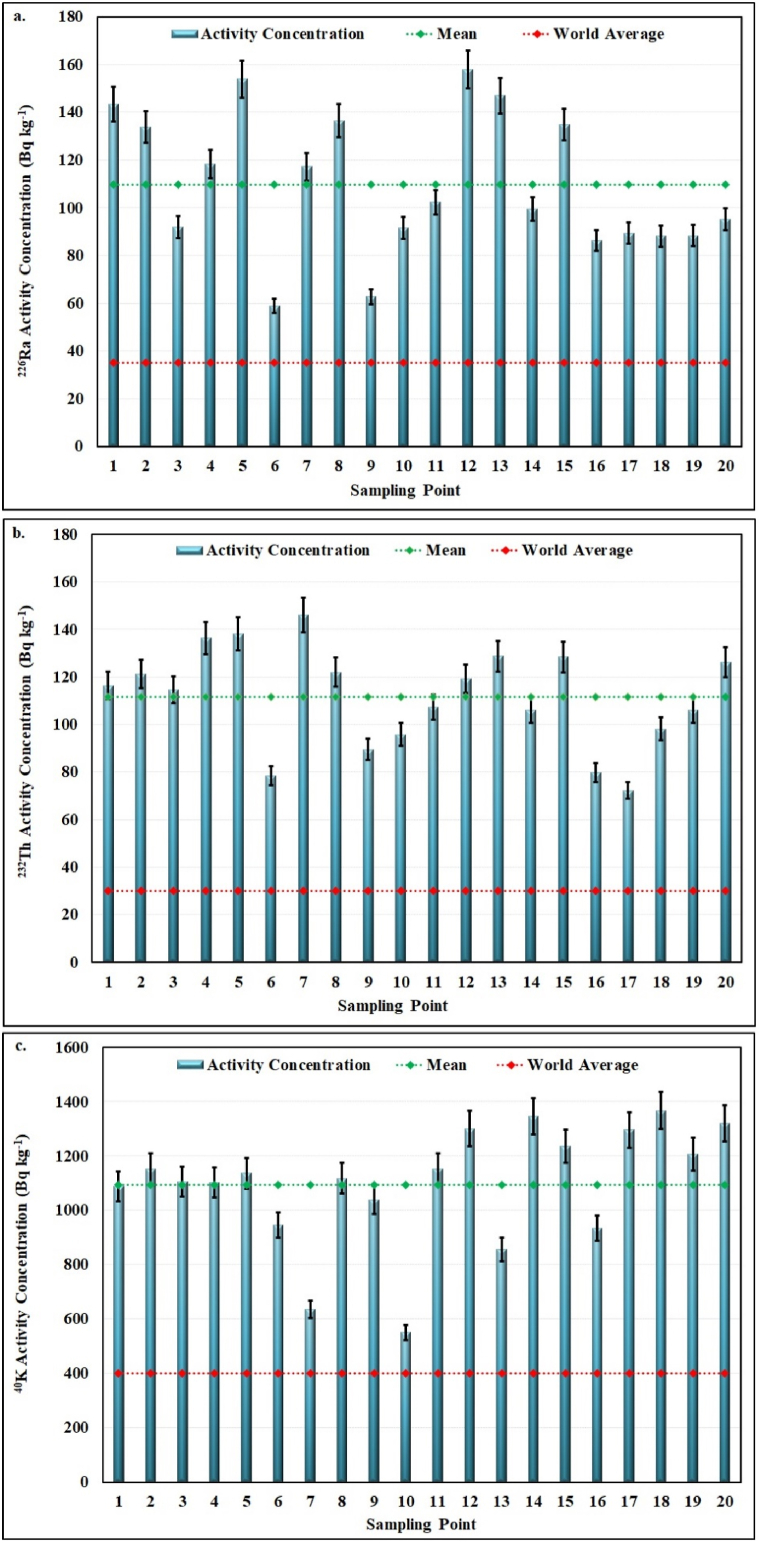
Graphical representation of activity concentrations of a. ^226^Ra, b. ^232^Th and c. ^40^K in soil samples.

Activity concentrations of ^226^Ra, ^232^Th and ^40^K in soil samples, mean of activity concentrations and world average values are shown in Fig. 2. The activity concentrations were found at a range of 58.95 ± 4.20–158.05 ± 19.95 Bq kg^−1^ for ^226^Ra (Figs. 2(a)), 72.28 ± 7.61–146.0 ± 22.27 Bq kg^−1^ for ^232^Th (Figs. 2(b)) and 550.76 ± 33.24–1367.50 ± 18.73 Bq kg^−1^ for ^40^K (Fig. 2(c)). The mean activity concentrations of ^226^Ra, ^232^Th and ^40^K in soil samples were calculated as 109.85 ± 10.44 Bq kg^−1^, 111.53 ± 8.38 Bq kg^−1^, and 1094.35 ± 35.55 Bq kg^−1^, respectively.

The measured mean values of ^226^Ra, ^232^Th and ^40^K were much higher than the reported worldwide permissible values of 35 Bqkg^−1^, 30 Bqkg^−1^ and 400 Bqkg^−1^, respectively [12]. As seen in Fig. 2, all values of analysed natural radionuclides are higher than the world average.

**Fig. 3:**
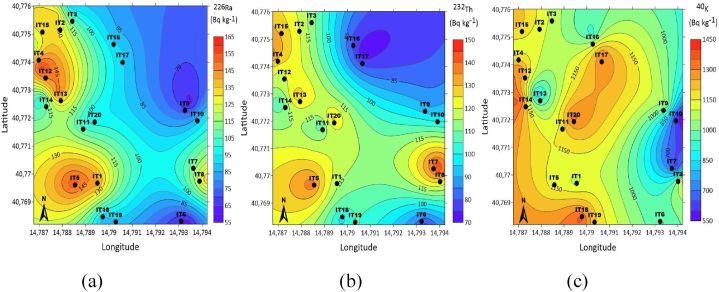
Distribution map of (a) ^226^Ra, (b) ^232^Th, (c) ^40^K activity concentration with sampling points and coordinates.

To determine the spatial distribution of natural radionuclides, radioactivity level distributions were determined using the Surfer 16 software program and displayed on radiological maps. The distribution maps of activity concentrations of ^226^Ra, ^232^Th and ^40^K in soil samples with sampling points and coordinates are given in Fig. 3.

Distribution maps of activity concentrations according to coordinates were obtained using the Kriging method in the Surfer 16 Program. Kriging is a method that uses data to predict and map variables in a certain region using the grid method [28]. Kriging allows estimating radiological values in the entire study area by including areas where experimental observations are not made [29]. On the distribution maps, classes are automatically formed based on the measured values form the highest activity concentration, in red, to the lowest ones, in blue.

As seen in Fig. 3(a), five distinct regions for ^226^Ra distribution map can be recognized. The activity concentrations of ^226^Ra were classified as low (55–100 Bq kg^−1^), moderate (100–125 Bq kg^−1^), high (125–130 Bq kg^−1^), and very high (135–145 Bq kg^−1^ and 145–165 Bq kg^−1^), which are blue, green, yellow, orange and red areas on the map. The lowest and the highest activity concentrations of ^226^Ra were obtained for the IT6 (40° 46′ 5.64″ N, 14° 47′ 36.14″ E) and the IT12 (40° 46′ 25.69″ N, 14° 47′ 13.47 ″E), respectively.

In Fig. 3(b), the activity concentrations of ^232^Th were classified by the Surfer program as low (70–100 Bq kg^−1^), moderate (100–120 Bq kg^−1^), high (120–125 Bq kg^−1^), very high (125–130 Bq kg^−1^ and 130–150 Bq kg^−1^), which are blue, green, yellow, orange and red areas on the map. The lowest activity concentration of ^232^Th was found for IT17 (40° 46′ 27.93″ N, 14° 47′ 26.33″ E) and the highest was found for IT7 (40° 46′ 13.06″ N, 14° 47′ 38.12″ E).

In the case of ^40^K, as can be seen in Fig. 3(c), the activity concentrations were classified as low (550–900 Bq kg^−1^), moderate (900–1100 Bq kg^−1^), high (1100–1150 Bq kg^−1^), very high (1150–1300 Bq kg^−1^ and 1300–1450 Bq kg^−1^), which are blue, green, yellow, orange and red areas on the map. The lowest and the highest activity concentrations of ^40^K were obtained for the IT10 (40° 46′ 19.69″ N, 14° 47′ 38.80″ E) and the IT18 (40° 46′ 6.29″ N, 14° 47′ 23.03″ E), respectively. Although activity concentrations are classified as moderate and low on radiological maps, they are at levels well above the world average.

A comparison of the activity concentrations of natural radionuclides in the present study in soil samples from Campania region, south of Italy, with other studies in different areas is presented in Table 1. The range of activity concentrations of all three investigated natural radionuclides, in this study, were found higher than the values for Upper Egypt, Kosovo, Erbil (Iraqi Kurdistan Region), Libya, Lithuania, Tunisia, and Skopje (Macedonia). The range values of activity concentration of ^40^K found is coherent with the other studies performed in different regions of the Italian peninsula. Activity concentration of ^226^Ra and ^232^Th are higher than the other italian results instead. In particular, the highest value of ^226^Ra content found in this study is lower only to results obtained in Korea as well as for ^232^Th. Since soil samples have different physical, chemical and geological structure and location characteristics, the results obtained are very site specific, varying depending on the region.

**Table 1:** Comparison of natural activity concentrations in this study with other studies around the world.

Study Area	Natural Activity Concentration (Bq kg^−1^)			References
	^226^Ra	^232^Th	^40^K	
Gudalore (India)	**-**	19–272	75–596	[30]
Upper Egypt	10–19	1–5	94–107	[31]
Kosovo	8–30	7–31	105–515	[32]
Serbia	20–55	30–73	167–559	[33]
Najran (Saudi Arabia)	9–41	8–49	203–993	[34]
Erbil (Iraqi Kurdistan Region)	10–16	9–11	242–342	[35]
Jordan	6–1134	MDA-168	19–1362	[36]
Korea	9–108	15–282	203–1560	[37]
Libya	**-**	5–17	242–424	[38]
Lithuania	2–37	0–21	155–710	[39]
Tunisia	5–50	5–30	93–319	[40]
Skopje (Macedonia)	24–42	38–52	502–707	[41]
Anatolian region of Istanbul (Turkey)	16–62	24–63	316–878	[11]
Northern Calabria (Italy)	11–29	–	336–1401	[42]
Lombardia (Italy)	**-**	20–70	242–1434	[43]
Campania (Italy)	59–158	72–146	551–1367	This study

The natural radioactivity levels are related to the geological conditions in an area, indeed. The activity concentrations of natural radionuclides in nature, clastic sediments and sedimentary rocks contain lower levels of activity concentration. Higher radionuclide concentrations are observed in rocks of volcanic origin and lower in sedimentary rocks [[44], [45], [46]]. In the Italian peninsula, in particular in the southern regions, there are many volcanic areas, so high radionuclide concentrations in the soil are expected.

### 3.2 Assessment of the impact on human health

**Fig. 4:**
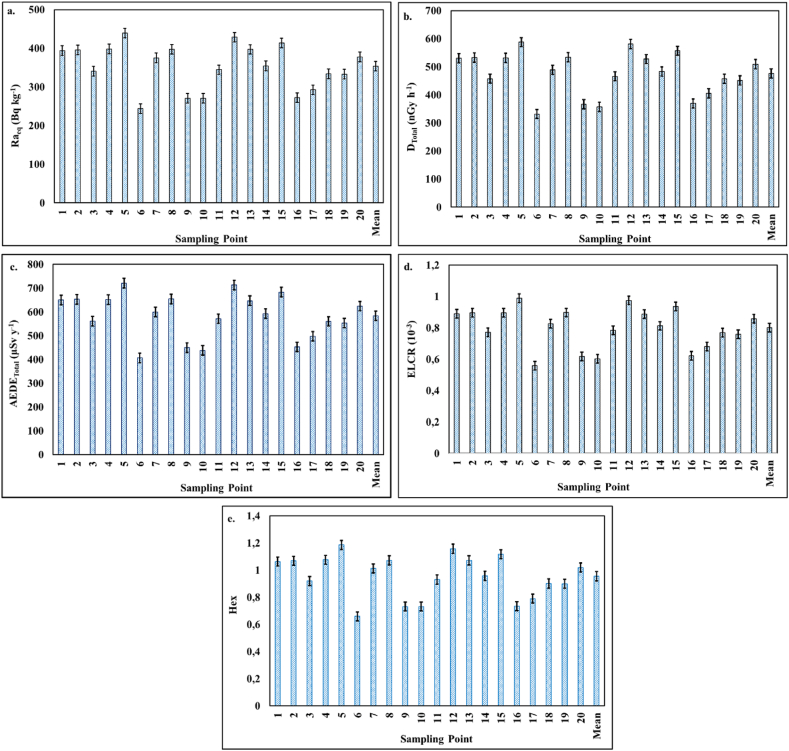
Charts of estimated health hazard indices: a. ${Ra}_{eq}$, b. *D*_*tota*l_, c. *AEDE*_*total*_*,* d. *ELCR* and e. *H*_*ex*_ estimates.

The absorbed gamma dose rate (*D*), the annual effective dose rate (*AEDE*), the radium equivalent activity (*Ra*_*eq*_), the external hazard index (*H*_*ex*_), and excess life cancer risk (*ELRC*) were calculated, for each soil sample, to assess the radiological health risk. The calculated radiological hazard indices graphs are given in Fig. 4.

The estimated *Ra*_*eq*_, due to the activity concentrations of natural radionuclides in soil samples at the sampling site, ranges from 244.11 to 438.93 Bq kg^−1^ as reported in Fig. 4(a). The mean value of *Ra*_*eq*_ is 353.60 Bq kg^−1^ which is slightly lower than the recommended permissible value of 370 Bq kg^−1^ [12]. *D* values vary from 114.13 to 201.92 nGy h^−1^ as indicated in Fig. 4(b).

The mean values of *D*_*out*_ and D_in_ are found to be 163.75 and 312.38 nGy h^−1^, respectively. The total absorbed dose rate (*D*_*tota*l_) ranged from 331.40 to 587.49 nGy h^−1^. The values of *AEDE*_*out*_, *AEDE*_*in*_*, ELCR* and *H*_*ex*_ ranged from 139.97 to 247.63 μSv y^−1^, from 266.46 to 472.86 μSv y^−1^, from 0.56x10^−3^ to 0.99x10^−3^, and from 0.66 to 1.19, respectively, as reported in Fig. 4(c–e). The total annual effective dose equivalent (*AEDE*_*total*_) ranged from 406.42 to 720.50 μSv y^−1^. The world average values of *D*_*out*_, *D*_*in*_, *AEDE*_*out*_, *AEDE*_*in*_, and *ELCR* are 59 nGy h^−1^, 84 nGy h^−1^, 70 μSv y^−1^, 340 μSv y^−1^ and 0.29x10^−3^, respectively [47]. The average values of *AEDE*_*out*_, *AEDE*_*in*_ and *ELCR* were found to be 200.82 μSv y^−1^, 383.10 μSv y^−1^ and 0.80x10^−3^, respectively, and these average values are higher than the world average values.

All the estimated parameters/indicators are higher than the world average values, indicating a high exposure to natural radionuclides, which in turn increases the likelihood of cancer in humans depending on the duration of exposure. The fact that radiological values are higher than the permitted values threaten human health in this region. *H*_*ex*_ value was found between 0.66 and 1.19. At sampling points, some values are higher than permissible value (1) and therefore there is a possibility that radiological hazard may occur.

Furthermore, the average absorbed dose rates, annual effective dose equivalent and excess lifetime cancer risk due to high radionuclide activity concentrations are higher than the permissible values worldwide.

#### 3.2.1 Statistical features

The statistical characteristics of the estimated parameters can be better analysed by using the third- and fourth-order statistical moments, skewness and kurtosis.

Skewness allows one to obtain information about the asymmetry in the distribution of a real-valued random variable. The kurtosis, instead, is likely to be the most effective property for identifying deviations from the normal (Gaussian) distribution. A symmetric Gaussian process is characterized by kurtosis equal to 0. Leptokurtic distributions (k > 0) can be associated to large outliers and/or heavy tails, whereas platykurtic distribution (k < 0) underlines an almost flat shape and/or lighter tails. Uniform distribution is a limit example. Some applications to environment, especially in seismological context, can be found in Ref. [22].

**Fig. 5:**
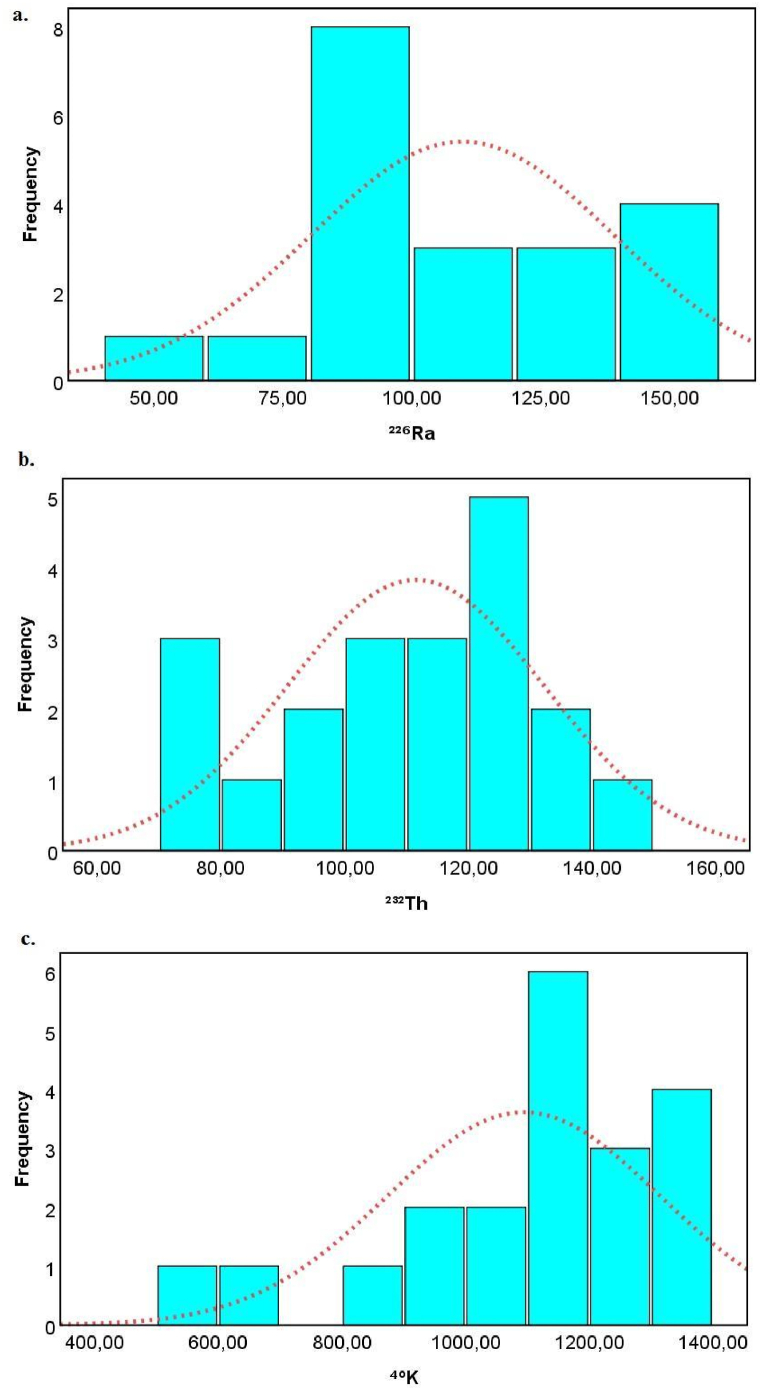
The frequency distributions of the activity concentrations of a. ^226^Ra, b. ^232^Th, and c. ^40^K in the soil samples.

The statistical analyses were performed by using IBM SPSS version 25.0 for Windows to evaluate the behaviour of activity concentrations and radiological parameters, and the relationships between variables [48]. The frequency distributions of the activity concentrations in the soil samples are given in Fig. 5. The skewness coefficient of activity concentration of ^226^Ra has approximately null value (0.09), which shows the distribution peak is nearly symmetric as seen in Fig. 5(a). The skewness coefficient of activity concentration of ^232^Th was found to be −0.36 as shown in Fig. 5(b). Since this value is between −0.5 and 0, providing indication of a moderately skewed curve. Finally, the ^40^K frequency distribution curve was found to be left skewed or negatively skewed [49], having estimated a negative skewness equal to −1.13 (Fig. 5(c)).

Kurtosis of activity concentrations of ^226^Ra, ^232^Th and ^40^K distribution curves were found to be −1.03, −0.68 and 1.08, respectively. The negative values for ^226^Ra, ^232^Th showing lighter tails. For ^40^K, the distribution is leptokurtic because the kurtosis is greater than zero, which means that the tail of the distribution curve is full of data [20,49]. In other words, the positive value of kurtosis indicates a long-tailed distribution ascribable to outliers.

To determine the relationship between variables and strength of association between pairs of variables, linear Pearson correlation coefficients for soil samples were calculated. Pearson's correlation matrix is given in Table 2. As seen in the matrix, there is a high positive correlation coefficient (0.730) between ^232^Th and ^226^Ra, but weak correlation coefficients (0.080 and −0.051) between ^40^K and other radionuclides. The reason for the weak correlation coefficients between ^40^K and other radionuclides is that the ^40^K radionuclide does not belong to the ^232^Th and ^238^U chains. *Ra*_*eq*_, *D*, *AEDE*, *ELCR* and *H*_*ex*_ have high good positive Pearson's correlation coefficients (r ≥ 0.841) with ^232^Th and ^226^Ra which indicates that these radionuclides contribute significantly to the calculated radiological hazard parameters. In addition, although the ^40^K radionuclide has a lower correlation coefficient with radiological parameters compared to other radionuclides, it has a weak positive correlation (0.308), which shows that the ^40^K radionuclide also poses a radiological hazard. Pearson correlation analysis suggested that natural radioactivity in the study area was due mostly to ^232^Th and ^226^Ra activity concentrations and somewhat ^40^K.

**Table 2:** Pearson's correlation matrix between couples of variables.

Variables	^226^Ra	^232^Th	^40^K	Ra_eq_	D	AEDE	ELCR	H_ex_
^**226**^**Ra**	1.000	0.730	0.080	0.908	0.898	0.898	0.898	0.908
^**232**^**Th**	0.730	1.000	−0.051	0.871	0.841	0.841	0.841	0.871
^**40**^**K**	0.080	−0.051	1.000	0.308	0.368	0.368	0.368	0.308
**Ra**_**eq**_	0.908	0.871	0.308	1.000	0.998	0.998	0.998	1.000
**D**	0.898	0.841	0.368	0.998	1.000	1.000	1.000	0.998
**AEDE**	0.898	0.841	0.368	0.998	1.000	1.000	1.000	0.998
**ELCR**	0.898	0.841	0.368	0.998	1.000	1.000	1.000	0.998
**H**_**ex**_	0.908	0.871	0.308	1.000	0.998	0.998	0.998	1.000

**Fig. 6:**
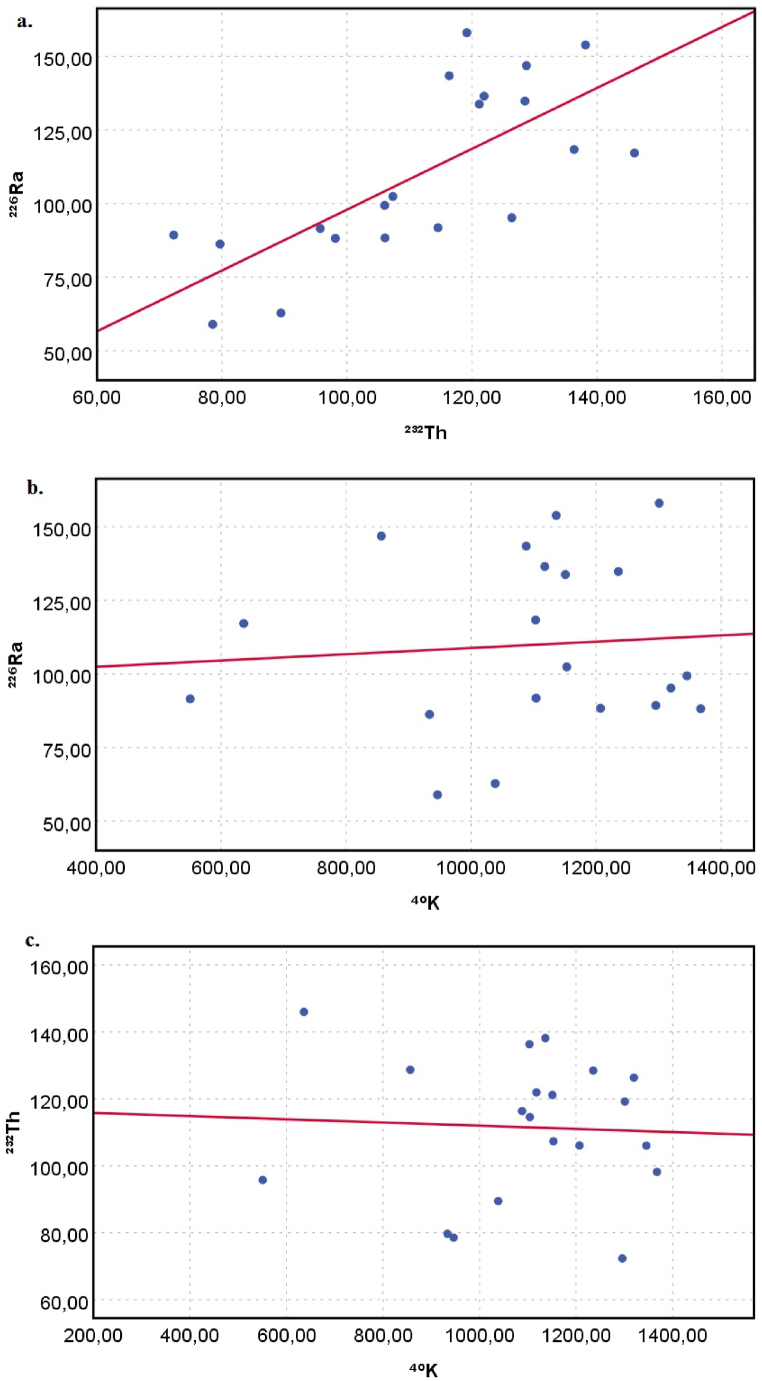
Correlation between a. ^226^Ra and ^232^Th, b. ^226^Ra and ^40^K, and c. ^232^Th and ^40^K.

Correlations between the combinations of radionuclides ^226^Ra and ^232^Th, ^226^Ra and ^40^K, ^232^Th and ^40^K are shown in Fig. 6(a–c). As seen from the correlation graphs (Fig. 6(a)), it is clear that a significant correlation was found between ^226^Ra and ^232^Th combination (R = 0.730, R^2^ = 0.533), confirmed by the high value of the Pearson correlation coefficient. Albeit a very low collation was found between both ^226^Ra and ^40^K (R = 0.080, R^2^ = 0.003) and ^232^Th and ^40^K (R = 0.051, R^2^ = 0.006), as indicated in Fig. 6(b and c), respectively. Nevertheless, it can be a feeble marker of geological and chemical characteristics [50].

### 3.3 Principal component analysis results

PCA was performed for radiological parameters using varimax rotation with the aim to extract those components (parameters) containing most information. The application of such technique to all the indicators provides the components listed in Table 3. According to the rotated factor loadings, component-1 and component-2 accounted for 83.3 % and 13.46 % of the total variance, respectively. The component-1 comprises ^226^Ra and ^232^Th with high loadings and all natural radionuclides have positive loadings. The radionuclides with high loadings in the component-1 are the most useful for individuating soil samples based on soil and rock characteristics [51]. Instead, no significant loadings were obtained for any variable of component-2, which is responsible for 13.46 % of the total variance. The results show that there are two eigenvalues and the total explained variance is 96.76 %. This value is much higher than 75 %, threshold above which the correlation results can be considered satisfying and reliable (see, e.g., Refs. [52,53]). Moreover, the total explained variance is very high meaning that the PCA results are in good agreement with Pearson correlation analysis.

**Table 3:** Rotated factor loadings of the radiological variables.

Variables	Components	
	1	2
^**226**^**Ra**	0.911	−0.189
^**232**^**Th**	0.867	−0.356
^**40**^**K**	0.309	0.949
**Ra**_**eq**_	1.000	−0.001
**D**	0.998	0.064
**AEDE**	0.998	0.064
**ELCR**	0.998	0.064
**H**_**ex**_	1.000	−0.001
**Variance in %**	83.30 %	13.46 %

## 4 **Conclusions**

Identifying all the elements of soil is vital to decision-making for environmental monitoring related to climate change, human activities, health risk assessment purposes etc. Urban soils can be object of redevelopment caused by human activities in the context of the evolution of the area, for example. Surely, the determination of the contents of the natural radionuclides ^226^Ra, ^232^Th, and ^40^K plays an important role in terms of health risk assessment but their presence in the environment could be considered also as an important database for a characterization in terms of background data of the soil since these radionuclides are naturally present in the earth crust in different amounts according to the original geological features of the subsoil. Aim of this study was to put in evidence how natural radionuclides could not only be monitored for radioprotection purposes but also could be taken into account in the characterization of urban top soils for the monitoring of possible land redevelopment. For this purpose, in particular, a spatial distribution methodology has been proposed and performed. So, the evaluation of the content and spatial distribution of NORM in the area of the university campus in the province of Salerno was performed, for the first time. The activity concentrations of natural radionuclide ^226^Ra, ^232^Th and ^40^K in 20 soil samples have been measured using high purity germanium (HPGe). The average ^226^Ra, ^232^Th and ^40^K activity in all the twenty samples were 109.85 ± 10.44 Bq kg^−1^, 111.53 ± 8.38 Bq kg^−1^, and 1094.35 ± 35.55 Bq kg^−1^, respectively. All measured ^226^Ra, ^232^Th, and ^40^K average activity concentrations exceeded the global averages. These high results reflect the volcanic characteristics of the investigated soils and, accordingly, all radiological hazard parameters are higher than the safe limit. This preliminary investigation may be useful to support further studies aimed at investigating more in-depth the contamination of the soil and the assessment of the radiological risk for the population living in the area and to put in evidence the necessity to include in the characterization of urban soil also the natural radionuclides content as ‘background indicators’. The spatial distribution could represent a basis for the identification of the background levels of natural contaminants of the area as well as for the definition of a precise model, at finer scale, of the distribution of radionuclides, thus allowing a better understanding of their origin, both natural (because of the presence of relevant volcanic structures) and anthropogenic along with their temporal evolution.

Indeed, screening information, such as the ^226^Ra, ^232^Th, and ^40^K contents database and spatial distribution, as started in this study, will allow for more focused time and cost-effective efforts when investigating and reviewing soil data from possible effects of human activities.

Further studies will be focused on the integration of the characterization of the soil with the measurements of artificial contaminants like ^137^Cs in the context of projects aimed to the study of new methodologies for the biodiversity and environmental preservation.

## Funding

Project funded under the National Recovery and Resilience Plan (NRRP), Mission 4 Component 2 Investment 1.4 - Call for tender No. 3138 of December 16, 2021, rectified by:

Decree n.3175 of December 18, 2021 of Italian Ministry of University and Research funded by the 10.13039/501100000780European Union – NextGenerationEU; Award Number: Project code CN_00000033, Concession Decree No. 1034 of June 17, 2022 adopted by the Italian Ministry of University and Research, CUP, H43C22000530001 Project title “National Biodiversity Future Center - NBFC”.

## Data availability statement

Data associated with this study have not been deposited into a publicly available repository. Data are included in article/supp. material/referenced in article.

## CRediT authorship contribution statement

**Serpil Aközcan Pehlivanoğlu:** Writing – review & editing, Resources, Methodology, Investigation, Data curation, Conceptualization. **Simona Mancini:** Writing – review & editing, Writing – original draft, Conceptualization. **Selin Özden:** Writing – original draft, Investigation, Formal analysis. **Michele Guida:** Supervision, Project administration. **Mariarosaria Falanga:** Writing – review & editing, Validation, Conceptualization.

## Declaration of competing interest

The authors declare that they have no known competing financial interests or personal relationships that could have appeared to influence the work reported in this paper.
