# Supplementary material for: Characterization of a typical urban soil in terms of natural radionuclide content. The case study of a university campus
Source: Heliyon. 2024 Aug 30;10(17):e37145. doi: 10.1016/j.heliyon.2024.e37145 (PMC11409136; doi:10.1016/j.heliyon.2024.e37145)
Supplement: Multimedia component 1 [file mmc1.docx]

**Table 1.** GPS coordinates of the sampling points.

| **Station number** | **GPS COORDINATES** | |
| --- | --- | --- |
|  | **^LATITUDE^** | **^LONGITUDE^** |
| 1 | 40.769716 | 40.769716 |
| 2 | 14.789487 | 14.789487 |
| 3 | 40.775685 | 40.775685 |
| 4 | 14.787760 | 14.787760 |
| 5 | 40.776015 | 40.776015 |
| 6 | 14.788325 | 14.788325 |
| 7 | 40.774505 | 40.774505 |
| 8 | 14.786772 | 14.786772 |
| 9 | 40.769653 | 40.769653 |
| 10 | 14.788443 | 14.788443 |
| 11 | 40.768234 | 40.768234 |
| 12 | 14.793372 | 14.793372 |
| 13 | 40.770294 | 40.770294 |
| 14 | 14.793922 | 14.793922 |
| 15 | 40.769788 | 40.769788 |
| 16 | 14.794219 | 14.794219 |
| 17 | 40.772545 | 40.772545 |
| 18 | 14.793537 | 14.793537 |
| 19 | 40.772137 | 40.772137 |
| 20 | 14.794111 | 14.794111 |

**Table 2.** Comparison of natural activity concentrations in this study with other studies around the world.

| **Study Area** | **Natural Activity Concentration (Bq kg^-1^)** | | | **References** |
| --- | --- | --- | --- | --- |
|  | **^226^Ra** | **^232^Th** | **^40^K** |  |
| Gudalore (India) | **-** | 18.8–272.1 | 77.5–595.9 | (Selvasekarapandian et al., 2000) |
| Upper Egypt | 10.5-18.7 | 1.5-4.6 | 94-107 | (El-Taher, 2011) |
| Kosovo | 8–30 | 7–31 | 105–515 | (Kadiri et al., 2022) |
| Serbia | 20.4-55.2 | 29.9-73.4 | 167-559 | (Milenkovic et al., 2015) |
| Najran (Saudi Arabia) | 8.67-41.54 | 8.56-49.09 | 202.85-993.07 | (Al-Zahrany and Al-Mogabes, 2013) |
| Erbil (Iraqi Kurdistan Region) | 10.6-16.2 | 8.8-10.7 | 241.8-340.9 | (Azeez et al., 2020) |
| Jordan | 6-1134 | MDA-168 | 19-1362 | (Alomari et al., 2019) |
| Korea | 9-108 | 15-282 | 203-1560 | (Yun et al., 2005) |
| Libya | **-** | 4.86- 17.05 | 242.4- 424.4 | (Alajeeli et al., 2019) |
| Lithuania | 2.1-37.4 | 0.3-21.3 | 155.3-710.1 | (Pečiulienė et al., 2020) |
| Tunisia | 5–50 | 5–30 | 93–319 | (Mahjoubi et al., 2006) |
| Skopje (Macedonia) | 24.1-41.9 | 38.5-52.2 | 502-707 | (Angjeleska et al., 2020) |
| Anatolian region of Istanbul (Turkey) | 16-62 | 24-63 | 316-878 | (Aközcan et al., 2021) |
| Northern Calabria (Italy) | 11-29 | - | 336-1401 | (Guagliardi et al., 2021) |
| Lombardia (Italy) | **-** | 20–70 | 242–1434 | (Guidotti et al., 2015) |
| Salerno (Italy) | 59-158 | 72-146 | 551-1367 | This study |


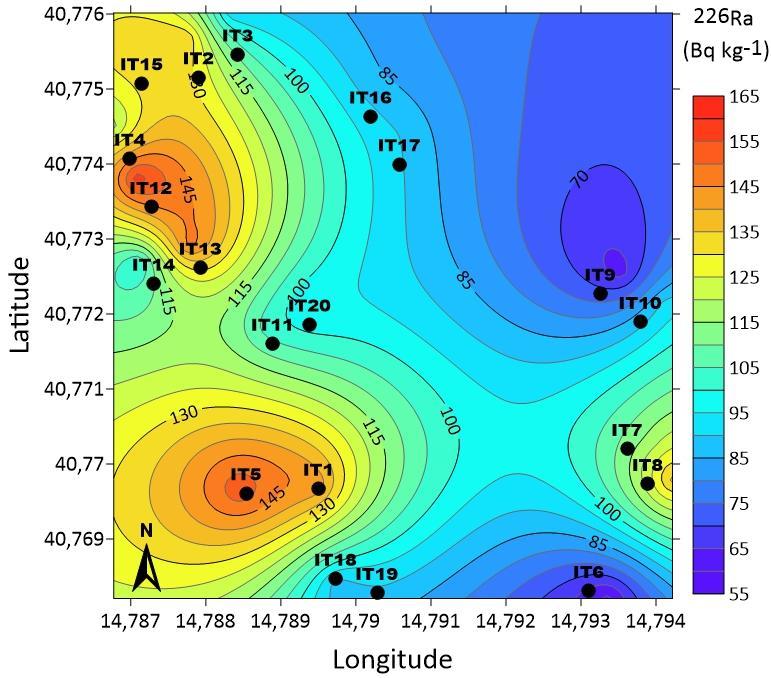


**Figure 3a.** Distribution map of ^226^Ra activity concentration with sampling points and coordinates.

**
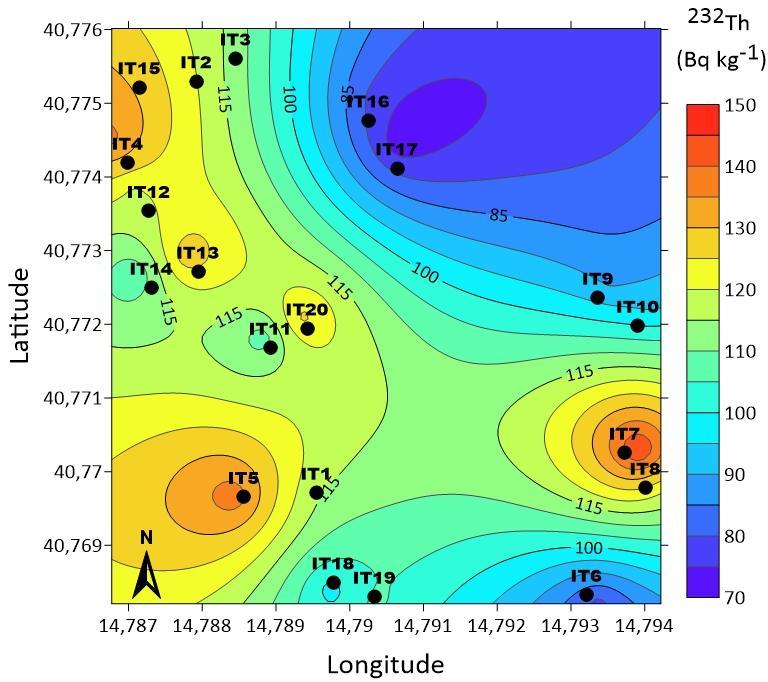
**

**Figure 3b.** Distribution map of ^232^Th activity concentration with sampling points and coordinates.

**
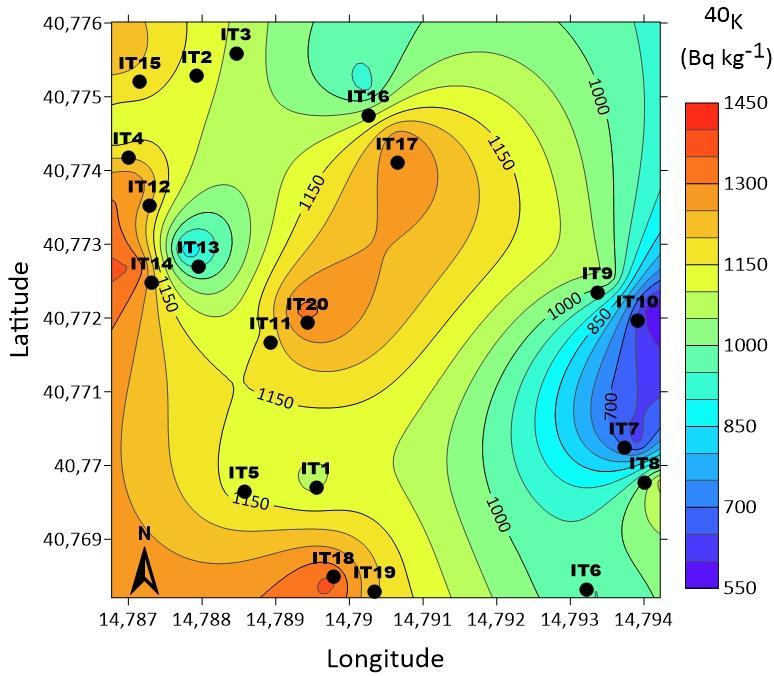
**

**Figure 3c.** Distribution map of ^40^K activity concentration with sampling points and coordinates.

**
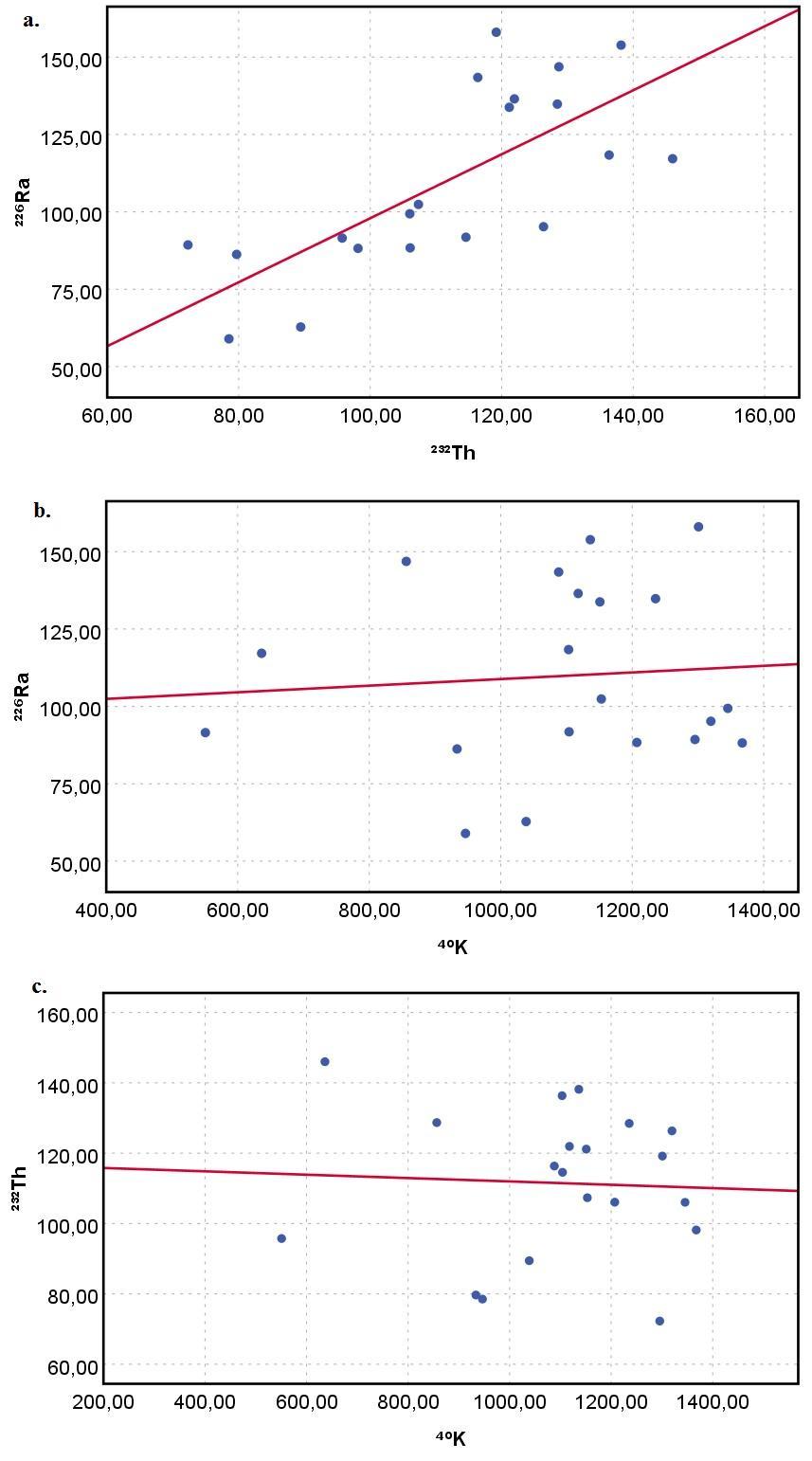
**

**Figure 4.** Correlation among a. ^226^Ra and ^232^Th; b. ^226^Ra and ^40^K; and c. ^232^Th and ^40^K.

**
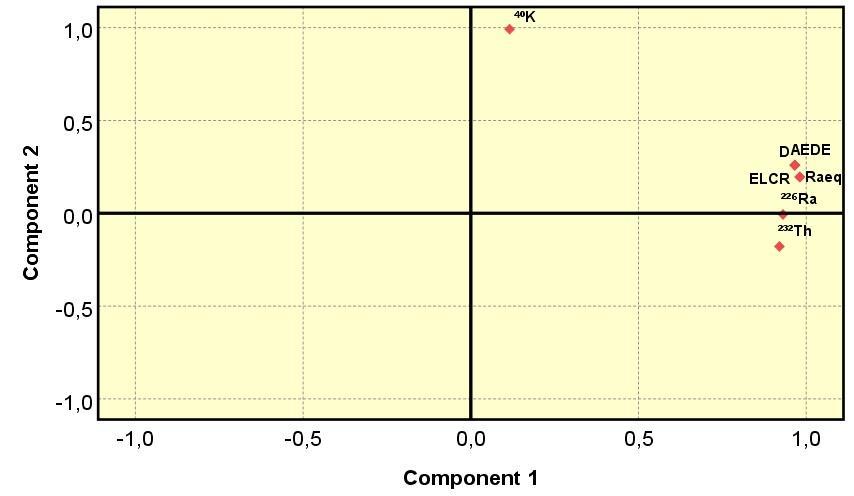
**

**Figure 5.** Plot of component-1 (83.30%) and component-2 (13.46%).
